# An audit to evaluate an institute’s lead researchers’ knowledge of trial registries and to investigate adherence to data transparency issues in an Italian research institute registry

**DOI:** 10.1186/s13063-018-2910-2

**Published:** 2018-09-20

**Authors:** Chiara Pandolfini, Maurizio Bonati

**Affiliations:** Department of Public Health, Laboratory for Mother and Child Health, IRCCS - Istituto di Ricerche Farmacologiche “Mario Negri”, via Giuseppe La Masa 19, 20156 Milan, Italy

**Keywords:** Randomized controlled trial (RCT), Statistics and research methods, Clinical trial registries, Clinical trial registration requirements, Ethical issues

## Abstract

**Background:**

Clinical trial registries have been a priority topic in the past few years in promoting data transparency and accountability. In this context, in 2011, the IRCCS - Istituto di Ricerche Farmacologiche “Mario Negri” set up a registry to collect data on all studies in which the institute’s researchers are involved. In this study we present a self-audit in order to detect the lead researchers’ general knowledge on registries, the completeness and quality of the randomized controlled trial (RCT) data inputted in an Italian research institute’s registry, and the researchers’ adherence to both registration requirements and the institute transparency goal, aiming to improve standards and leading to greater awareness of the issues involved.

**Methods:**

A questionnaire-based audit was conducted. To interview researchers we included questions ranging from general knowledge on registries (e.g., what are the aims of registries?) to questions about their knowledge of the Mario Negri’s registry, questions on selected trials and registration, included information on the protocol, and the results.

**Results:**

The audit sample covers 12 of the 47 RCTs at the institute’s Milan branch, representing all the possible lead researchers responsible for RCTs at the institute. The researchers have more than a basic knowledge of trial registries and their aims. All the researchers reported that they know of the ClinicalTrials.gov registry and most of them reported that they frequently use it; however, only a few know about the World Health Organization’s registry platform (International Clinical Trials Registry Platform). The most cited registry aims reported were increased transparency and reduced publication bias. Of the studies registered in the institute’s registry, 92% had at least one data item missing in the registry record. Concerning trial registration in the international registries, all 12 respondents said their trial had been registered and specified the registry name, but often they had not inputted the associated trial ID code in the corresponding field of the institute’s registry. Concerning two important issues on data transparency and ethical standards, namely registration timing and result reporting, 11 stated that their trial was registered before starting recruitment, and for five of six closed trials they stated that their results have been already published—for one trial within 1 year after its completion.

**Conclusions:**

Researchers should guarantee correct reporting of trials and their data as a rule of great ethical value. Institutional self-audits should be performed periodically in order to improve clinical trial disclosure.

## Background

Clinical trial (CT) registries are a priority topic because they contribute to promote data transparency and accountability. It is of relevance to disseminate CT registration requirements and awareness among researchers. Information on data quality and transparency is still suboptimal in CT registries, including outdated information [1] and biases, such as those related to publication and outcome reporting [2, 3]. Although over the years additional information has been added, including trial results, initiatives around the world have increasingly called for including in CT registries complete results reporting [4–6] as a mandatory registration requirement [7–11]. The World Health Organization (WHO) has recently made a statement placing considerable importance on results reporting and its timing, calling for including in the CT registries the main findings published in peer-reviewed journals within 12 months from study completion or otherwise making CT results publicly available within 24 months, and for the key outcomes to be posted within 12 months in the CT registry results section [12]. Although various studies found several discrepancies among trial results reported in registries and results of the same trials published in the scientific literature [13–15], we deem it important to add significant pressure on researchers’ accountability, so as to improve CT data transparency.

In 2011, the IRCCS - Istituto di Ricerche Farmacologiche “Mario Negri” in Milan, Italy, set up a registry to collect data on all the studies in which the institute’s researchers are involved as coordinators or participants, including experimental and observational trials [16]. With this registry, the institute aims to ensure that information on the research carried out by the institute is made publicly transparent and accessible to anyone in the public or in the local and international scientific communities, to promote trial registration, and to stimulate research collaboration among researchers. The registry collects data related to each of the 20 items in the WHO’s minimum data set requirements for CT registries [17]. It also collects additional data, such as age range of the population involved in the trial and bibliographic reference of publications related to the trial. In the Italian research institute registry the data are collected in English and Italian.

Considering the growing awareness related to CT registries and access to trial data, in order to raise pressure on researchers adherence to the new standards for transparency, undertaking audits, especially self-audits, seems to be the best way of increasing knowledge on CT registries in the scientific community [18]. Audits can be conducted on different aspects of healthcare and, through their application, individual physicians, institutions, pharmaceutical companies, or scientific communities can obtain the means to improve their care or research [19]. Auditing CT results reporting, for example, can lead to greater awareness of institution gaps in data transparency and can be used to improve standards [20]. Recently an audit study on pharmaceutical companies’ policies on transparency of trials and their concordance with ethical and professional guidance revealed a wide variation in following registration requirements [21]. We performed an audit to detect the general knowledge of experienced lead researchers on CT registries, the completeness and quality of the data inputted in the Mario Negri Italian research institute registry, and the researchers’ adherence to registration requirements aimed at ensuring the institute transparency goal.

## Methods

A questionnaire was created to interview researchers; it included questions ranging from general knowledge on CT registries (e.g., what are the aims of registries?), to knowledge of the Mario Negri registry, to questions on the selected trials and registration requirements, including information on CT protocols and results.

In May 2017 all researchers at the institute were asked to update their trial records, a reminder that is sent at least twice a year. The data on ongoing research studies and on studies completed at least 1 year before, present in the registry by 7 June 2017, were used for this study. At least two randomized controlled trials (RCTs) from each department based in the Milan branch of the institute registered in the Italian research registry were selected. Whenever possible, an open and a closed RCT were selected. The responsible researchers of these selected trials, preferably different medical doctors for each RCT, were then interviewed for the audit. The questionnaire was previously tested on two researchers to check the clarity of the questions; two questions needed to be made more explicit. We avoided involving these two researchers in the survey. The face-to-face interviews, carried out between the two authors and the researcher responsible for the selected studies, took place between 22 June and 24 October 2017.

The data included in the registry, especially those related to the WHO’s minimum data set, were also checked for completeness and compared, in some cases, to the replies given with the face-to-face interviews. We checked also whether the trials had been registered in the ClinicalTrials.gov registry. If the two authors were in doubt after the interviews, they reached an agreement on any uncertainty regarding the respondents’ answers.

## Results

A total of 188 studies were present in the registry, 128 of which were in the Milan branch (See Table 1). Of these, 47 were RCTs and 12 (26%) were covered by the audit sample. Four out of five Milan departments (except Environmental Health Sciences, which did not have any RCTs in the registry) were covered by the audit sample, as were all the researchers responsible for the 47 Milan RCTs (except MB, who could not participate because he is one of the authors and interviewers).

**Table 1:** Number of studies registered in the Mario Negri registry and numbers of features reported in the audit sample

	Italian Mario Negri registry	Milan branch registry	Audit sample
Studies in registry	188	128	12
RCTs	67	47	12
RCTs completed	33	23	6
RCTs running (open/interrupted)	34	24	6
People responsible for studies	38	30	12
People responsible for RCTs	17	13	12
Departments	9	5	4

The researchers seemed to have more than a basic knowledge of CT registries. All respondents declared that they know about ClinicalTrials.gov and 9/12 said that they use it often. The most common uses are to assess the state of the art of a research area (75%), to look for information on specific trials (42%), and to search for publications (33%). Only 67% of responders know that ClinicalTrials.gov also includes observational studies. One third (4/12) of researchers declare they know of the WHO’s International Clinical Trials Registry Platform (ICTRP).

When asked, in general, what the aims of clinical trial registries are, the most common aims were to increase transparency (75%), reduce publication bias (58%), promote the public accountability of medical research in general (50%), prevent unnecessary duplication of research effort, promote collaboration between researchers, and improve access to clinical trials (33% each).

All knew the Mario Negri registry; whereas 92% of respondents seemed sure that it includes experimental trials, another 75% said it also includes observational studies. Only two guessed the correct date when the registry was launched, namely in the year 2011. In terms of the aims of the institute’s registry, the most common aims were to increase transparency (100%) and/or visibility (75%). Less frequently reported aims were the following: to promote collaboration between researchers, to increase the public accountability of medical research, and to improve access to clinical trials (66% each).

Most of the studies (92%) had at least some information missing in the registry (mostly a trial’s short name or acronym). “Lack of time”, “we forgot”, and “I don’t remember” were the reasons for not having updated the data entered in the registry.

In only one study were the patients involved in protocol development, and only one of the six completed trials included a newsletter providing patients with informative material about the progress and results of the CT.

When researchers were asked if they had launched a press release concerning the CT, eight replied “no”, two “did not know”, and two said they had done so at the end of the study to disseminate results.

All 12 respondents said their trial had been registered in the large, internationally acknowledged registries: EudraCT for 10 trials and ClinicalTrials.gov for 8 (see Table 2 for details). Concerning time of registration, 92% stated that their trial was registered before recruitment began, and one did not know when the trial was registered.

**Table 2:** Registration of the 12 RCTs reported in acknowledged registries and trial result publication. A few audit replies to the proposed questionnaire and registry data comparison

Number rec	Status	If the trial is closed, have the results been published?	Has the reference of the published article been added in the registry?	Time from the closing of the trial to the publication (years, months)	In which trial registry was the study registered?	Reported trial registry and registration ID in published paper
1	Closed	Yes	No	7 m	EudraCT and ClinicalTrials.gov	Yes
2	Closed	No	(In press)	–	EudraCT and ClinicalTrials.gov	–
3	Closed	Yes	Yes	4 y, 4 m	EudraCT (and maybe also ClinicalTrials.gov)	No
4	Closed	Yes	No	1 y, 2 m	ClinicalTrials.gov	Yes
5	Closed	Yes	Yes	2 y, 9 m	EudraCT and ClinicalTrials.gov	Yes
6	Closed	Yes	Yes	1 y, 4 m	EudraCT and ClinicalTrials.gov	Yes
7	Interrupted	Yes	Yes	–	EudraCT	Yes
8	Open	–	–	–	ClinicalTrials.gov	–
9	Open	–	–	–	EudraCT and ClinicalTrials.gov	–
10	Open	–	–	–	EudraCT	–
11	Open	–	–	–	EudraCT and ClinicalTrials.gov	–
12	Open	–	–	–	EudraCT	–

Regarding publication of the protocols, only three had been published, according to respondents (two before recruitment and one afterwards, all in international scientific journals). Five of six closed trials had already published the results, and one was in press. One CT was published within 1 year of its completion.

## Discussion

The researchers seem to have a good overall awareness of the aims of CT registries in general. The presence of the WHO’s important initiative and the ICTRP platform grouping together the major registries worldwide is less well known.

The importance given to the Mario Negri registry, in terms of completeness and update status of the entered data, is weaker than expected. This may be due to the fact that researchers have limited time and resources to dedicate to inputting data in registries, so they focus on reporting data where it is mandatory, as is the case for Europe with the EudraCT registry and for the US ClinicalTrials.gov registry [8, 22].

Most of the studies had at least some data corresponding to the WHO’s 20 minimum data items in the registry, showing that some of the information deemed fundamental for trial transparency by the WHO is underestimated by all researchers. In May 2017 the WHO decided to include information that was considered important for the trial registries to collect: details of the ethics committees approving each trial, the study completion dates, and results availability [23]. Since about half of CTs continue to remain unpublished, one hopes that this additional information will give an additional boost to data transparency [23]. The Mario Negri registry aims to collect all the WHO trial requirements data. However, concerning results availability, data completeness and details on the publication of results is suboptimal in the registry. Conversely, given that trials on drug interventions remain largely unpublished, it is a positive result that, although it constitutes a small sample, all the trials concluded at the Mario Negri Institute are being published [24].

Concerning the timeliness of CT publication, legislative or simply authoritative initiatives have called for CT results publication within 1 year [8, 12, 25], but, as found elsewhere [26, 27], the portion of trials published in accordance with the initiatives is limited (one out of six of the completed trials evaluated in this audit was published within 1 year). It is difficult for researchers, usually with limited resources, to comply with publication of results in scientific journals, which is often due to the long journal peer review times as found in this audit. More importantly, briefly reporting results data in plain language in trial registries might overcome the delay in published data availability, thus providing a quick dissemination in scientific and lay communities. Unexpectedly, in our audit sample researchers of only two completed trials disseminated trial results via press release, thus limiting information on possibly important information through large public media.

Concerning trial registration, as required by the EU legislation, all drug intervention trials are registered in EudraCT. Almost all the trials are also registered in ClinicalTrials.gov, although respondents rarely recall it. The related ID number is often unreported in the Mario Negri registry. Although not mandatory for already registered trials, the high percentage of the Mario Negri trials registered also in ClinicalTrials.gov is likely due to the great pressure from journal editors [28] wishing to know results and increase awareness on transparency, hence possibly disseminating important issues from research protocols. Complying with these editor requirements, all the six trials completed at the Mario Negri Institute mentioned the ID registration numbers in the published papers. This positive finding allows others to identify the trial in multiple registries, to detect publications of the same protocols thus avoiding data duplication, accurately calculate effect size in meta-analyses, and reduce bias and over-represented results in systematic reviews.

Some limits to this study should be noted. The audit was performed on a small sample of researchers; thus, the findings are not completely representative of the level of knowledge on the aims and importance of CTs globally generalizable to other scientific institutions. The researchers interviewed, however, represent all possible lead researchers responsible for the institute’s CTs and therefore represent an advanced context in terms of knowledge in running of CTs. In addition, the Mario Negri registry is a local registry and does not have a link in the WHO ICTRP registry [17]; therefore, its data entries are visible only to researchers searching for this specific registry [29].

The results of this audit show that there is still much to do to raise awareness of the importance of registries, especially since the researchers involved are lead researchers. Additional information on the importance of registries could, in general, help raise awareness and could improve the registry’s data, thus increasing the institute transparency level.

## Conclusions

Reporting clinical research accurately is of potential interest to researchers, healthcare professionals, and patients. Improving the transparency and quality of reporting in clinical research is one of the ethical obligations that researchers should satisfy. CT reporting and data transparency can influence the uptake of scientific research findings into policy or practice.

CT information and results should be publicly disseminated as part of the heritage of the whole community to aid in community well-being. Further regulatory policy and editor efforts might publicly award those clinical researchers keen on accessing, using, and adding data in their registered protocols.

## Abbreviations

CT: Clinical trial
ICTRP: International Clinical Trials Registry Platform
RCT: Randomized clinical trial
